# Differential regulation of 5-lipoxygenase and leukotriene-C4-synthase expression by IFNγ, IL-4 and IL-13 in human monocytes and macrophages from patients with atopic dermatitis

**DOI:** 10.1007/s00011-025-02108-2

**Published:** 2025-11-26

**Authors:** S. Lietzau, S. Traidl, C. Riesselmann, T. Werfel, S. Mommert

**Affiliations:** Department of Dermatology and Allergy, Hannover Medical School, Carl- Neuberg-Strasse 1, 30625 Hannover, Germany

**Keywords:** Atopic dermatitis, Macrophages, Single-cell RNA sequencing Th1 /Th2 cytokines, Biosynthesis of cysteinyl leukotrienes

## Abstract

**Introduction:**

Skin lesions in atopic dermatitis (AD) are characterized by elevated levels of both Th1 and Th2 cytokines, along with increased concentrations of inflammatory mediators such as cysteinyl leukotrienes (CysLTs). The enzymes 5-lipoxygenase (5-LO/ALOX5) and leukotriene-C4-synthase (LTC4S), which are essential in CysLT biosynthesis, are highly expressed by macrophages. We aimed to investigate the expression of 5-LO/ALOX5 and LTC4S mRNA in lesional AD skin at single cell level. Furthermore, we analyzed the regulatory effects of the Th1 cytokine interferon gamma (IFNγ) and the Th2 cytokines IL-4, IL-13 on 5-LO/ALOX5 and LTC4S expression in monocytes and macrophages derived from AD patients and healthy volunteers.

**Methods:**

Single-cell RNA sequencing (scRNA-seq) data from lesional AD skin biopsies, as reported in a previously published study, were re-analyzed to evaluate 5-LO/ALOX5 and LTC4S mRNA expression. PBMCs from AD patients, healthy volunteers and anonymous donors were used to isolate monocytes. Macrophages were generated in the presence of GM-CSF or M-CSF for 10 days. Cells were stimulated with IFNγ, IL-4 or IL-13. 5-LO/ALOX5 and LTC4S mRNA expressions were quantified by q-PCR. Intracellular 5-LO/ALOX5 expression was assessed by immunocytochemistry.

**Results:**

Re-analysis of scRNA-seq data revealed high levels of 5-LO/ALOX5 and LTC4S transcripts in monocytes and macrophages. In-vitro, IFNγ induced 5-LO/ALOX5 mRNA expression in blood derived monocytes and macrophages from AD patients, and protein levels in both monocytes and macrophages from anonymous donors, whereas IL-4 and IL-13 suppressed its expression. Vice versa LTC4S mRNA expression was downregulated by IFNγ but upregulated by IL-13. Higher baseline mRNA expressions of 5-LO/ALOX5 and LTC4S were observed in blood derived monocytes from AD patients compared to cells from healthy volunteers .

**Conclusion:**

We demonstrate that IFNγ enhances the 5-LO/ALOX5-catalyzed stage of CysLT synthesis, whereas IL-13 promotes the LTC4S dependent pathway in human monocytes or macrophages. These findings suggest a potential role for these cells in driving CysLT production in acute and chronic AD lesions and may give rise for future therapeutic interventions targeting this pathway.

**Graphical abstract:**

Single-cell RNA sequencing (scRNA-seq) data from lesional atopic dermatitis (AD) skin biopsies, as reported in a previously published study, were re-analyzed to assess the expression of 5-lipoxygenase (5-LO/ALOX5) and leukotriene-C4-synthase (LTC4S). In parallel, peripheral blood mononuclear cell (PBMC) derived monocytes from AD patients, healthy volunteers (HV), and anonymous donors were differentiated into macrophages in the presence of either M-CSF or GM-CSF, and the expression of 5-LO/ALOX5 and LTC4S was evaluated. The major enzymes involved in cysteinyl leukotriene (CysLT) generation are 5-LO/ALOX5 (activated by the 5-LO/ALOX5-activating protein FLAP), which converts arachidonic acid to leukotriene A4 (LTA4) and LTC4S, which catalyzes the conjugation of LTA4 with glutathione to form leukotriene C4 (LTC4). LTC4 is subsequently metabolized extracellularly to LTD4 and LTE4. ScRNA-seq analysis revealed that both 5-LO/ALOX5 and LTC4S were highly expressed in monocyte/macrophage clusters. Specifically, 5-LO/ALOX5 was strongly expressed in GM-CSF–differentiated (M1-like) macrophages, while LTC4S expression was upregulated in M-CSF–differentiated (M2-like) macrophages. These patterns were confirmed in blood-derived macrophages from anonymous donors. Interferon gamma (IFNγ) induced 5-LO/ALOX5 mRNA expression in blood-derived monocytes and macrophages from AD patients, as well as 5-LO/ALOX5 protein in cells from anonymous blood donors. LTC4S mRNA expression was upregulated by interleukin 13 (IL-13) in cells from HVs. Monocytes from AD patients displayed higher baseline mRNA levels of 5-LO/ALOX5 and, more strikingly, of LTC4S compared with cells from HVs. NS = non-stimulated.
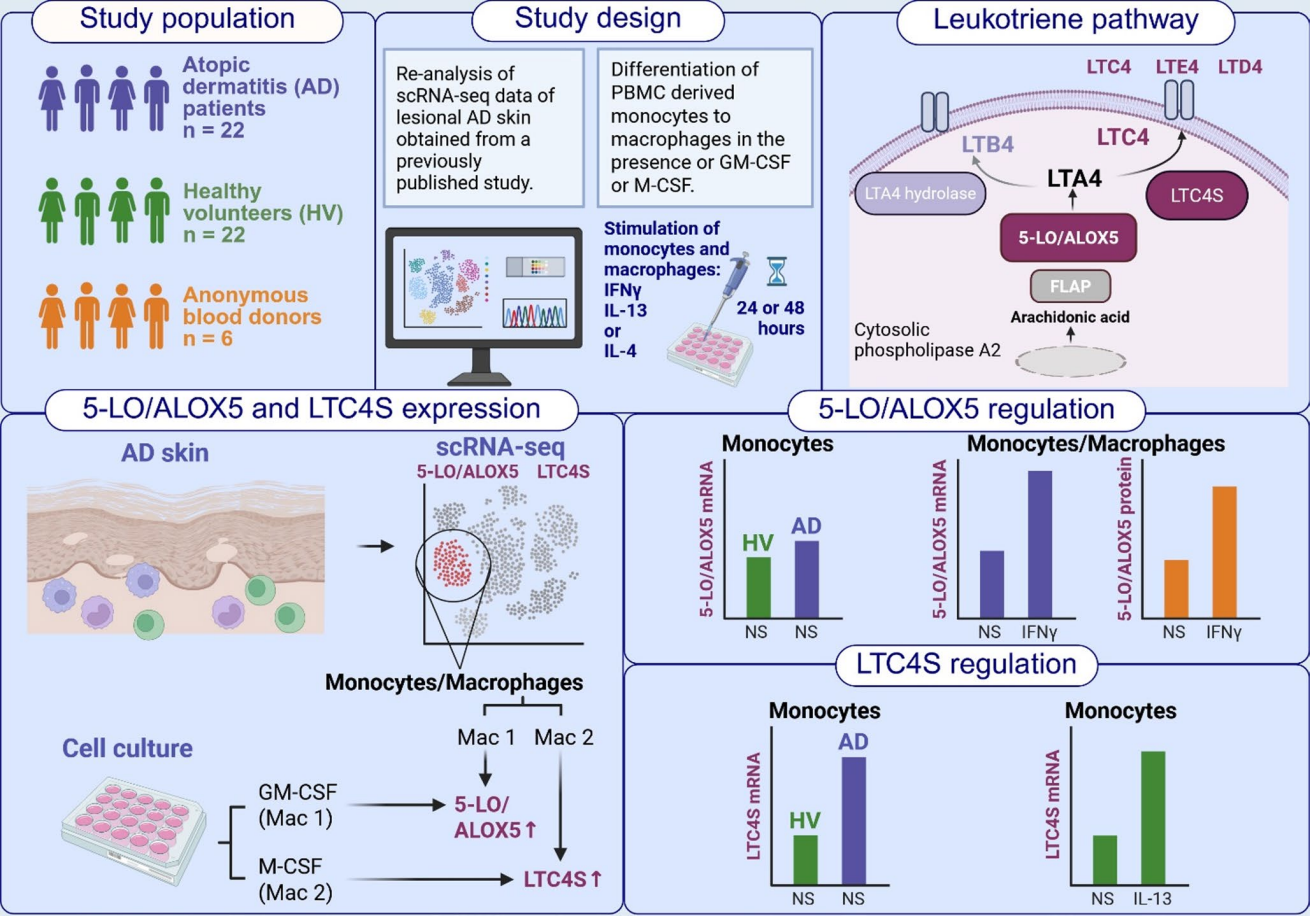

## Introduction

Atopic dermatitis (AD) is a complex chronic inflammatory skin disease characterized by skin barrier dysfunction and an imbalanced immune system. AD manifests as eczema, dry skin, and pruritus, significantly impacting patients’ quality of life. AD presents in acute and chronic phases, with distinct clinical features. Acute lesions are dominated by Th2 cells producing IL-4 and IL-13, while chronic lesions are additionally characterized by a Th1 cell response with IFNγ production [1–3].

Recent single-cell RNA sequencing results from lesional AD skin have revealed that the disease-specific T-cell clusters are predominantly of the Th2/Th22 subpopulation [4]. Overexpression of Th2 cytokines leads to skin barrier impairment by downregulating key proteins essential for the stability of the stratum corneum and by reducing the production of antimicrobial peptides [5].

Cysteinyl leukotrienes (CysLTs) were recently proposed to contribute to the pathogenesis of AD as well [6]. CysLTs are derived from arachidonic acid (AA) and released during a wide range of inflammatory processes in different kinds of tissues. The major enzymes involved in CysLT production are the 5-lipoxygenase (5-LO/ALOX5), synthesizing leukotriene A4 (LTA4) from AA and the leukotriene-C4-synthase (LTC4S). LTC4S catalyzes the conversion of LTA4 and glutathione into the CysLT C4 (LTC4) [7].

Elevated levels of LTC4 were found in the skin of AD patients compared to healthy volunteers [8]. Studies conducted on mouse models have shown that LTC4 plays a role in inducing hallmark features of AD. LTC4 contributes to hyperkeratosis, a characteristic feature of AD involving thickening of the epidermis [9]. LTC4 has also been shown to be a potent inducer of itch [10, 11].

In both acutely and chronically inflamed AD skin lesions an accumulation of macrophages has been identified by the expression of the macrophage differentiation marker CD68 and the scavenger receptor CD163 [12]. The cytokines IFNγ, IL-4 or IL-13 in AD skin affect cell metabolism and macrophage polarization [12, 13].

Data regarding the influence of the inflammatory milieu of AD on the regulation of the cysteinyl leukotriene system are currently missing. Since macrophages highly express the enzymes required for CysLT synthesis, our research focused on the effects of Th1 and Th2 cytokines, which are known to play a crucial role in AD, on the expression of 5-LO/ALOX5 and LTC4S in monocytes and macrophages from AD patients and from healthy volunteers.

## Materials and methods

### Single-cell RNA sequencing

Data from our previous study including full thickness inflamed skin samples from 10 AD patients were used [4]. Briefly, reads obtained from the chromium chip (10x Genomics, Pleasanton, CA, USA) were aligned to the human reference genome GRCH38 by the CellRanger pipeline version 3.1.0. UMAP, feature plot and dot plot were created based on the expression matrix using the Seurat package version 4.2.3 [14].

### Isolation of monocytes and differentiation of macrophages

Healthy volunteers (HVs) (White, n = 22, 9 females and 13 males mean age 35.3 years) and patients with moderate-to-severe extrinsic atopic dermatitis (AD) who did not receive systemic treatment (White, n = 22, 14 females and 8 males mean age 35.6 years) were recruited from the Department of Dermatology and Allergy, Hannover Medical School, Hannover, Germany. AD was diagnosed according to the criteria of Hanifin and Rajka [15]. Venous blood samples were taken from both groups.

For the experiments showing the differential expression of 5-LO/ALOX5 and LTC4S mRNA in monocytes and macrophages, PBMCs were isolated from anonymous residual blood samples obtained during routine platelet collection at the Department of Transfusion Medicine, Hannover Medical School, Hannover Germany.

PBMCs were separated by density gradient centrifugation on lymphoprep (Fresenius Kabi Norge AS, Oslo, Norway). With a seeding density of 1 × 10^6^ cells per well, PBMCs were plated in a 24-well plate in Iscoves Medium supplemented with Human Serum AB male (PAN Biotech GmbH, Aidenbach, Germany) (2.5% v/v). To attach the monocytes, cells were incubated for 2 h at 5% CO_2_ at 37 °C. To ensure a high purity of the cells, non-adherent cells were removed by vigorously washing of adherent cells three times with PBS. Monocytes were incubated in an appropriate amount of RPMI 1640, supplemented with 2 mM l-glutamine, 100 mg/mL penicillin/streptomycin, 12 mM Hepes, and 5% v/v Human Serum AB male (PAN Biotech GmbH, Aidenbach, Germany; all other media components from Biochrom, Berlin, Germany).

Macrophage cultures were additionally supplemented with 10 ng/mL granulocyte macrophage colony-stimulating factor (GM-CSF) for M1 polarisation (Mac 1) or macrophage colony-stimulating factor (M-CSF) for M2 polarisation (Mac 2) (R&D, San Diego, CA, USA) and incubated for 5 days. On day 5, another 50% by volume of fresh medium containing GM-CSF/M-CSF was added. On day 8, the medium was completely changed. After 10 days, macrophages appear as adherent cells with a prominent nucleus and multiple pseudopodia. The human macrophages used in our experiments were of high purity. Any non-adherent cells that may have remained in the medium were effectively removed during the 8-day cultivation period by repeated medium changes and thorough washing. In addition immunocytochemistry images (Fig. 4A and C) of monocytes and macrophages, cultured on chamber tec slides, showed no other immune cell types except of monocytes or macrophages.

In our previous publication [16] using macrophages generated according to the similar protocol, intracellular staining revealed a significant upregulation of the differentiation marker CD68, while extracellular staining showed a trend toward increased expression of the scavenger receptor CD163 (indicative of M2 polarization) in M-CSF—differentiated macrophages compared to monocytes. Of note, in the present study we did not explicitly work with predefined M1 or M2 macrophages. Instead, we focused on GM-CSF– and M-CSF–differentiated macrophages, which in the unstimulated state do not yet display the typical M1 or M2 marker profiles, but mainly express the macrophage differentiation marker CD68.

### Stimulation of monocytes and macrophages

Monocytes and macrophages were stimulated with IL-4 (20 ng/mL), IL-13 (15 ng/mL) or IFNγ (200 ng/mL) (all R&D Systems, Wiesbaden, Germany) for 24 h and 48 h for analyzing the mRNA expression and 24 h for analyzing the protein expression by immunocytochemistry. The stimulatory doses of the cytokines for in vitro activation of macrophages were developed by our research group through extensive experiments that consistently elicited robust responses to IL-4, IL-13, and IFNγ in various immune cell types. The selected concentrations were published from our research group and others [17–19]. Afterwards cells were collected in RNA-stabilizing lysis buffer or RIPA Lysis and Extraction Buffer (Thermo Scientific™, Bremen, Germany).

### Quantitative real time (RT)-PCR

Total RNA was isolated using the innuPREP kit Micro RNA kit (Analytik Jena, Jena, Germany) according to the manufacturer’s instructions. The cDNA was synthesized by reverse transcription (QuantiTect reverse transcription kit, Qiagen, Hilden, Germany). Quantitative RT-PCR (q-PCR) was performed according to the MIQE guidelines with Quantitect^®^ primer assays for 5-Lipoxygenase (5-LO/ALOX5) (QT00015337), leukotriene-C4-synthase (LTC4S) (QT00000266), and RPS20 (ribosomal protein S20) (QT00003290) using SYBR^®^ Green according to the manufacturer’s instructions (Qiagen, Hilden, Germany) using the LightCycler (LC) 480. The amount of the target mRNA relative to the amount of the reference gene mRNA RPS20 (Target/Reference ratio (Tgt/Ref)), in the same sample was calculated by the Software LC 480 (Roche Molecular Biochemicals, Mannheim, Germany).

### Immunocytochemistry

For immunocytochemistry, PBMCs were isolated from anonymous residual blood samples obtained during routine platelet collection at the Department of Transfusion Medicine, Hannover Medical School, Hannover, Germany. Monocytes and macrophages (1.5 × 10^4^ cells per chamber) were cultured and differentiated as described above using growth chambers called chamber tek slides (Sarstedt AG & Co. KG, Nümbrecht, Germany), which were specially developed for microscopy of in situ cell culture of adherent cells. The chambers were removed, and the monocytes or macrophages on the microscope slide were fixed with 4% formaldehyde and processed for immunocytochemistry. The microscope slides were overlaid with pre-determined optimal concentrations of mouse IgG2a anti-5-LO/ALOX5 antibody (Cloud-Clone Crop., Katy, USA (5 µg/mL)) and in parallel with the corresponding isotype IgG2a in the same final concentration as a control. Detection was performed for the primary mouse antibody with the Dako EnVision kit HRP-APC (Dako, Agilent Technologies, Santa Clara, USA) according to the manufacturers’ instructions. Finally, the sections were counterstained by H&E staining according to common methods and mounted in Roti-Mount Aqua (Roth, Karlruhe, Germany). Microscopy was performed with the Pannoramic Digital Slide Scanner Midi II (3D Histech, Budapest, Hungary). On whole slide scans total cells and 5-LO/ALOX5 positive cells were counted using the QuPath software (QuPath, Queen's University Belfast, Belfast, Nordirland, UK).

### Statistics

For statistical analyses, the software GraphPad Prism version 8.0 was used (GraphPad Prism software, San Diego, CA, USA). First, we performed the D’Agostino-Pearson omnibus normality test, the Anderson-Darling test, the Shapiro-Wilk normality test and the Kolmogorov-Smirnov normality test to assess the assumption of a normal distribution in the data. In all our experiments, due to the individual variations of the data, the normality tests failed. The nonparametric test Wilcoxon matched-pairs signed-rank test was used to test whether the medians of two independent samples are different. For nonparametric unpaired samples the Mann-Whitney test was applied. Friedman Dunn’s Multiple Comparison test was used for paired group comparisons, Kruskal-Wallis test was used for unpaired group comparisons. The medians are shown in the graphs. A *p*-value < 0.05 was regarded as statistically significant (*p* < 0.05 was labelled with *, *p* < 0.01 was labelled with **, *p* < 0.001 was labelled with ***, *p* < 0.0001 was labelled with ****).

## Results

### Single-cell RNA sequencing of lesional AD skin reveals expression of 5-lipoxygenase (5-LO/ALOX5) and leukotriene-C4-synthase (LTC4S) mRNA most prominent in human monocytes and macrophages

We re-analyzed a data set obtained from AD patients in our previous publication through single-cell RNA sequencing (scRNA-seq) on immune cells enriched from lesional skin biopsies [4], with a specific focus on the expression of 5-LO/ALOX5 and LTC4S. Our goal was to define the role of these leukotriene pathway enzymes in AD and to pinpoint the cell populations with the highest expression and likely functional relevance of 5-LO/ALOX5 and LTC4S.

**Fig. 1:**
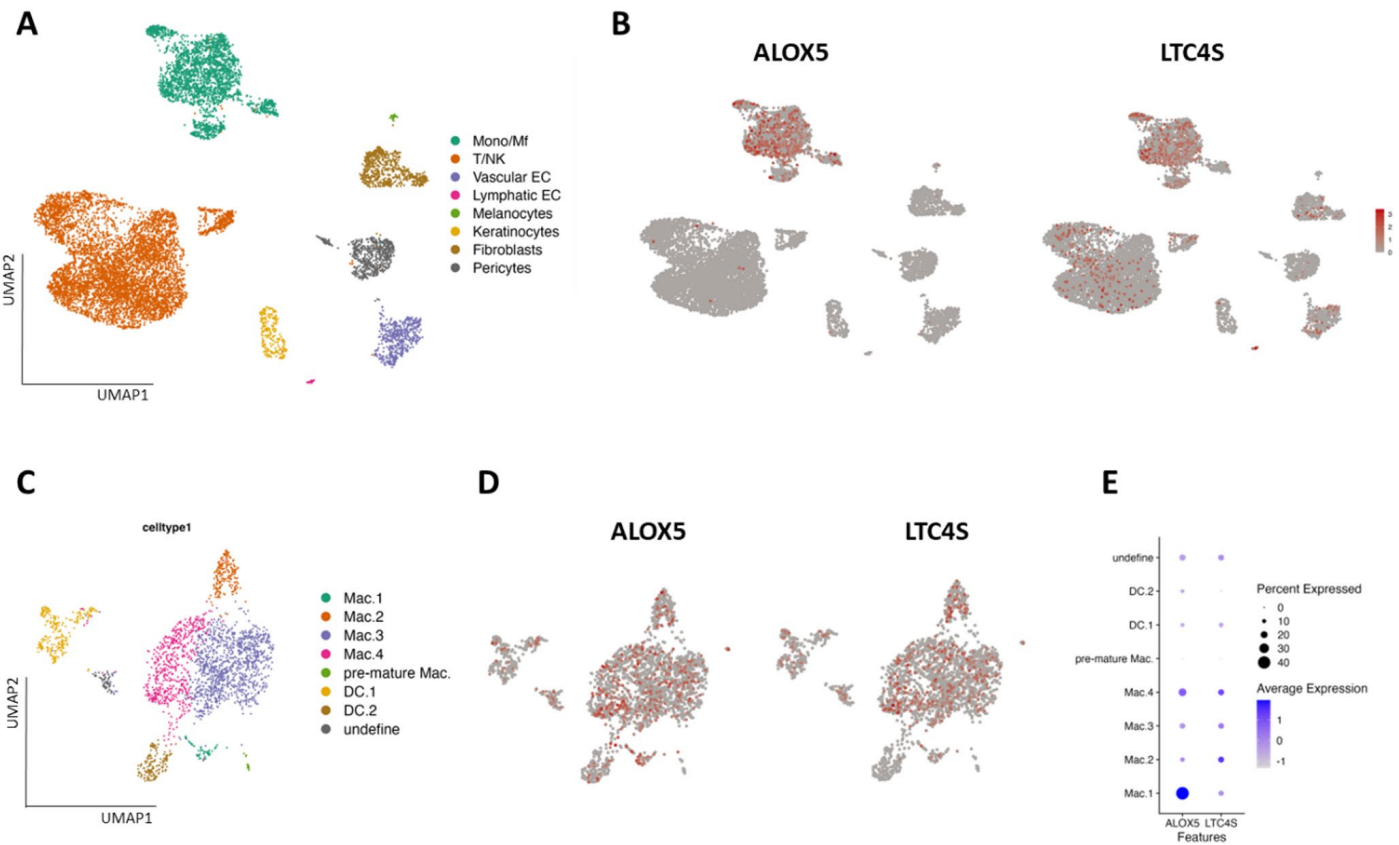
In skin from atopic dermatitis (AD) patients 5-Lipoxygenase (5-LO/ALOX5) mRNA and Leukotriene-C4-synthase (LTC4S) mRNA are mainly expressed in monocytes and macrophages. **A** UMAP visualisation (analyzed by Zhang et al.) showing different cell clusters in skin biopsies obtained from lesional inflamed AD skin: Mono = Monocytes, Mf = Macrophages, T = T cells, NK = Natural killer cells, EC = Endothelial cells, **B** 5-LO/ALOX5 and LTC4S mRNA expression in the different cell clusters depicted by UMAP. **C** Subsets of monocytic cells: Mac = Macrophages, pre-mature Mac = Monocytes, DC = Dendritic cells. **D** 5-LO/ALOX5 and LTC4S mRNA expression in the monocyte macrophage subsets. **E** Dot plot showing expression of 5-LO/ALOX5 and LTC4S in premature Mac (monocytes), different dendritic cell types (DC) and in macrophage (Mac) subsets. MRNA expression was evaluated by single-cell RNA sequencing

Based on cell type-specific marker gene expression, Zhang et al. identified not only major immune cell populations but also non-immune cell types, including endothelial cells, melanocytes, keratinocytes, fibroblasts, and pericytes [4] (Fig. 1A).

We detected pronounced expression of 5-LO/ALOX5 mRNA and LTC4S mRNA in the monocyte and macrophage cluster (Mono/Mf) relative to other cell populations. Additionally, LTC4S mRNA was also expressed at low levels in T cells, natural killer cells (T/NK), fibroblasts and endothelial cells (EC) (Fig. 1B).

To improve resolution within the Mono/Mf cluster, Zhang et al. [4] further subclassified macrophages into distinct populations: one characterized by high mRNA expression of classical (M1-like) pro-inflammatory markers (Mac.1; TNF, IL1B, IL18); another expressing markers associated with alternatively activated (M2-like) macrophages (Mac.2; CD163, CCL13, CCL17, CCL18); and two closely related macrophage subsets (Mac.3 and Mac.4) defined by high IFITM3 expression and differential IL1B expression. The dataset also included distinct dendritic cell (DC) populations and a small undefined cluster with mixed marker profiles (Fig. 1C) [4].

Our data analysis revealed that both 5-LO/ALOX5 and LTC4S mRNA were expressed in the Mono/Mf cell cluster, as shown in Fig. 1D.

Notably, a dot plot comparing myeloid subpopulations revealed that M1-like macrophages (Mac.1) exhibited the highest expression of 5-LO/ALOX5 mRNA, while LTC4S mRNA expression was more pronounced in M2-like macrophages (Mac.2) and in the Mac.4 subset highlighting functional heterogeneity in leukotriene pathway gene expression across macrophage subtypes (Fig. 1E).

However, scRNA-seq analysis of lesional atopic dermatitis skin biopsies did not detect mRNA expression of 5-LO/ALOX5 or LTC4S in keratinocytes. This is consistent with published data which show that expression of these enzymes in keratinocytes is variable and often very low or undetectable under native conditions, but can be induced in culture [20, 21]. Overall, both our scRNA-seq results and previous studies indicate that immune cells are the main and functionally relevant sources of 5-LO/ALOX5 and LTC4S in most physiological and pathological settings [22].

### Differential expression of 5-LO/ALOX5 and LTC4S mRNA in monocytes and macrophages cultured in the presence of GM-CSF or M-CSF with elevated baseline expression levels in monocytes from AD patients

**Fig. 2:**
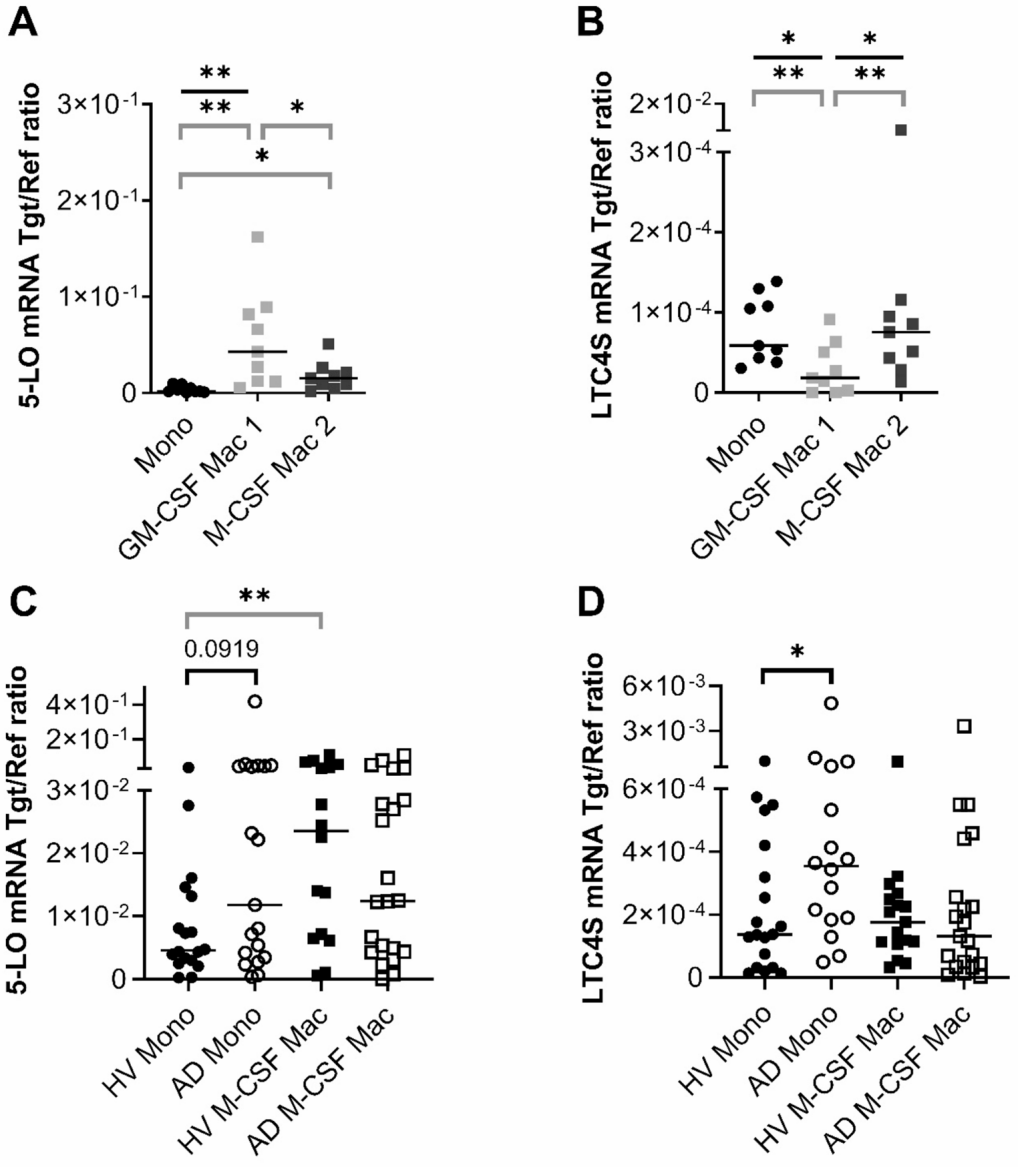
Differential expression of 5-LO/ALOX5 and LTC4S mRNA in monocytes and macrophages cultured in the presence of GM-CSF or M-CSF with elevated baseline expression levels in monocytes from AD patients. RNA was extracted from human monocytes after 24 h, from GM-CSF Mac. 1 and M-CSF Mac. 2 after 10 days. The cells were isolated from PBMCs from anonymous donors (**A**, **B**) and from healthy volunteers (HV) and AD patients (**C**, **D**). 5-LO/ALOX5 baseline mRNA expression in monocytes and macrophages (**C**) is shown as pooled data from non-stimulated cells (NS) from Figures 3A+B and Figures 3C+D. LTC4S baseline mRNA expression in monocytes and macrophages (**D**) is shown as pooled data from non-stimulated cells (NS) from Figures 5A+B and Figures 5C+D. 5-LO/ALOX5 (**A**, **C**) and LTC4S mRNA (**B**, **D**) expressions were detected by q-PCR and shown as target/reference (Tgt/Ref) ratio. Data are shown as individual values with medians. Significant differences, as determined by Friedman Dunn´s multiple comparison test (black straight bars), Wilcoxon matched-pairs signed rank test (grey angled bars) or Mann-Whitney U Test (black angled bars) are indicated as follows: *p < 0.05; ** p < 0.01; **A** and **B** (n = 9); **C** (n_HV_ Mono = 18, n_AD_ Mono = 19, n_HV_ Mac = 16, n_AD_ Mac = 22); **D** (n_HV_ Mono = 19, n_AD_ Mono = 16, n_HV_ Mac = 17, n_AD_ Mac = 21)

To validate the findings from scRNA-seq analysis, we isolated monocytes from anonymous blood donors. Macrophages were differentiated in the presence of GM-CSF or M-CSF. RNA was extracted from monocytes after 24 h and from macrophages after 10 days. Quantitative PCR analysis showed low expression of 5-LO/ALOX5 mRNA in monocytes. GM-CSF-macrophages exhibited significantly higher 5-LO/ALOX5 mRNA compared to both M-CSF-macrophages and monocytes (Fig. 2A). In contrast, the LTC4S mRNA expression pattern differed markedly: GM-CSF-macrophages showed lowest expression of LTC4S mRNA. In monocytes and M-CSF-macrophages LTC4S mRNA expression was significantly upregulated compared to GM-CSF-macrophages (Fig. 2B). However, when interpreting the results, the limited sample size must be considered. These results support a distinct regulatory pattern for 5-LO/ALOX5 and LTC4S mRNA expression in the different macrophage phenotypes and monocytes detected in lesional AD skin by scRNA-seq (Fig. 1E).

**Fig. 3:**
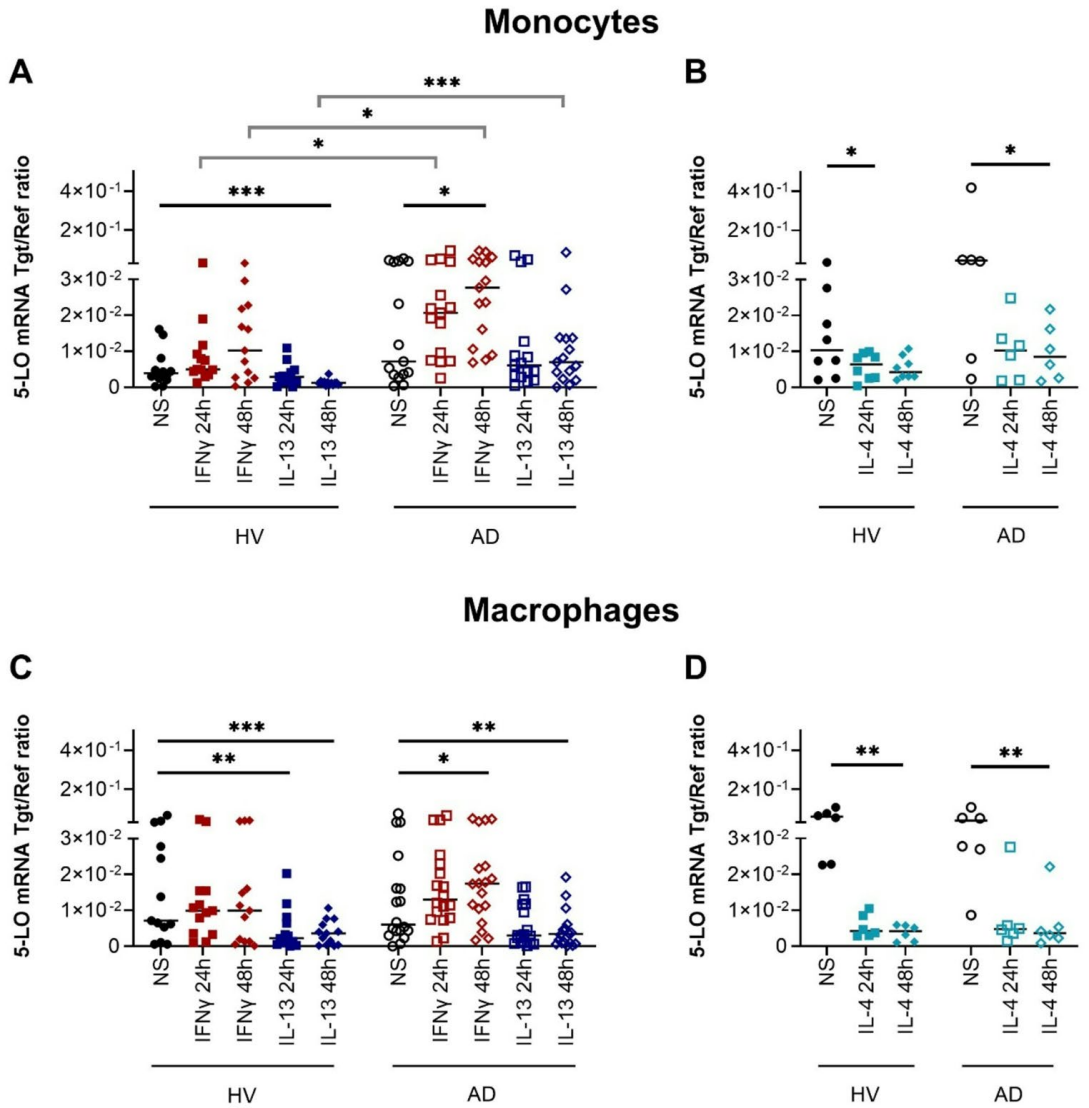
5-Lipoxygenase (5-LO/ALOX5) mRNA expression is upregulated by IFNγ and downregulated by IL-4 and IL-13 in human monocytes and macrophages from atopic dermatitis (AD) patients. Human monocytes (**A**, **B**) and macrophages differentiated in the presence of M-CSF (**C**, **D**) obtained from healthy volunteers (HV) and from AD patients were stimulated with IFNγ, IL-13 or IL-4 for 24 and 48 h or left non-stimulated (NS). 5-LO/ALOX5 mRNA expression was detected by q-PCR and shown as target/reference (Tgt/Ref) ratio. Data are shown as individual values with medians. Significant differences, as determined by Friedman Dunn´s multiple comparison test (black straight bars) or by Kruskal-Wallis test (grey angled bars) are indicated as follows: *p < 0.05; **p < 0.01; ***p < 0.001; **A** (n_HV_ = 13, n_AD_ = 15); **B** (n_HV_ =  8, n_AD_ = 6); **C** (n_HV_ = 13, n_AD_ = 18); **D** (n_HV_ = 6, n_AD_ = 6)

To investigate whether endogenous factors in AD patients influence the baseline expression of 5-LO/ALOX5 and LTC4S, we compared monocytes and M-CSF-macrophages from AD patients and healthy volunteers (HVs), using pooled data from Figs. 3 and 5. We detected a trend toward a higher baseline expression of 5-LO/ALOX5 mRNA and a significantly elevated baseline expression of LTC4S mRNA in monocytes from AD patients compared to HVs (Fig. 2C and D). In contrast, baseline mRNA expression levels of both 5-LO/ALOX5 and LTC4S were nearly identical in M-CSF-differentiated macrophages derived from monocytes of the same donors in both groups (Fig. 2C and D).

### 5-Lipoxygenase (5-LO/ALOX5) mRNA expression is upregulated by IFNγ and downregulated by IL-4 and IL-13 in human monocytes and macrophages from AD patients

To investigate the regulation of 5-LO/ALOX5 expression, we isolated monocytes and differentiated macrophages in the presence of M-CSF. To experimentally mimic the microenvironment of acute and chronic AD in an experimental setting, we stimulated both cell types with Th2 cytokines IL-4, IL-13 or the Th1 cytokine IFNγ, respectively.

We measured the mRNA expression of 5-LO/ALOX5 which catalyzes the conversion of arachidonic acid to leukotriene A4 (LTA4). LTA4 serves as a precursor for the generation of leukotrienes [7]. We observed significant upregulation of 5-LO/ALOX5 in response to IFNγ in monocytes and in macrophages obtained from AD patients when compared to cells from HVs. Notably, IL-13 downregulated 5-LO/ALOX5 mRNA expression in monocytes from HVs and in macrophages from HVs and AD patients. This suppressive effect was more pronounced in monocytes and macrophages from HVs when compared to AD patients (Fig. 3A and C). Significantly higher levels of 5-LO/ALOX5 mRNA expression were detected in IFNγ and IL-13 stimulated monocytes from AD patients compared to cells from HVs (Fig. 3A). IL-4 also reduced 5-LO/ALOX5 mRNA expression in human monocytes and macrophages from HVs and from AD patients (Fig. 3B and D).

### 5-Lipoxygenase (5-LO/ALOX5) protein expression is upregulated by IFNγ in human monocytes and macrophages

**Fig. 4:**
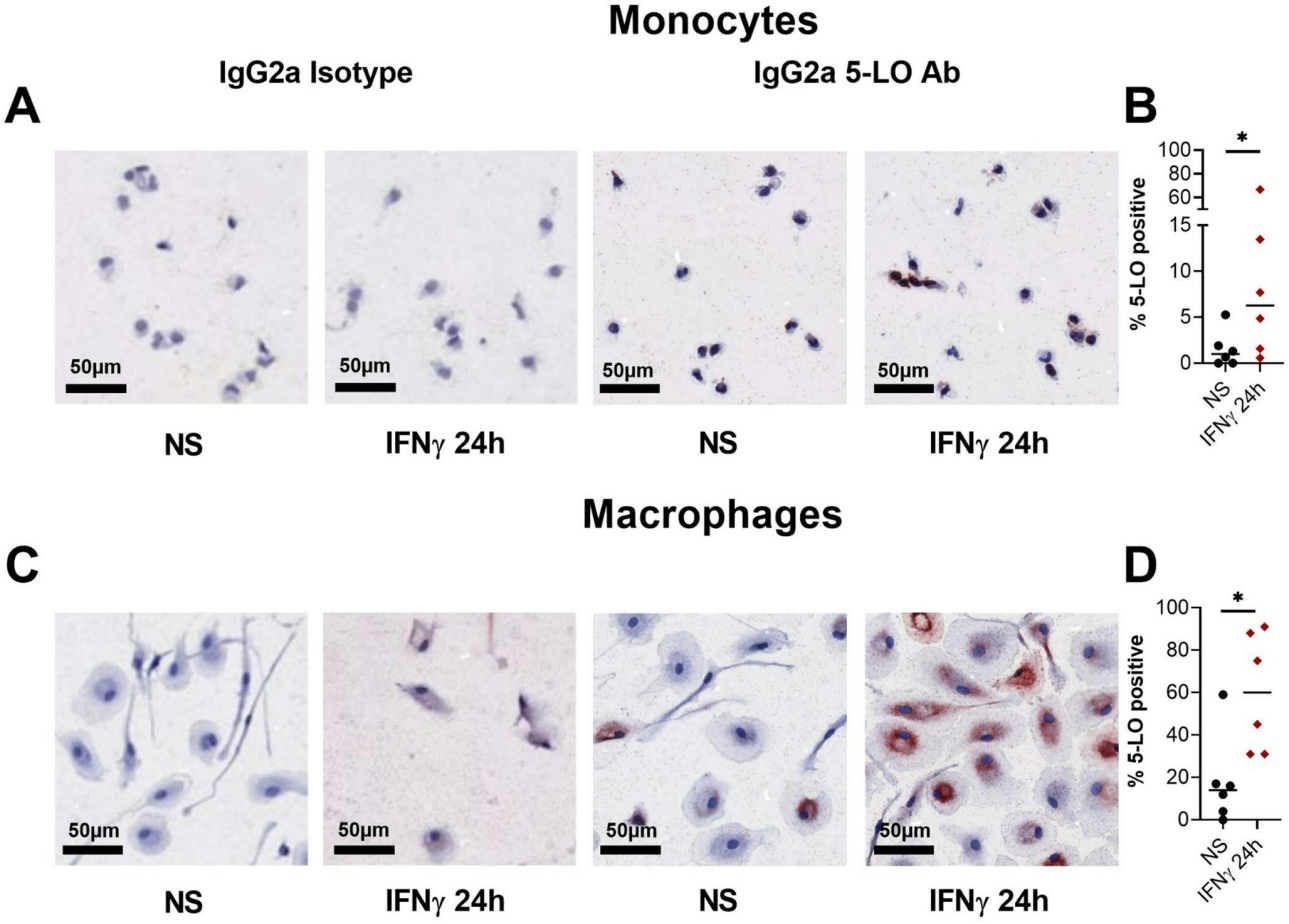
5-Lipoxygenase (5-LO/ALOX5) protein expression is upregulated by IFNγ in human monocytes and macrophages. Monocytes (**A**) and macrophages (**C**) were cultured in growth chambers (chamber tek slides) with IFNγ for 24 h or left non-stimulated. Immunocytochemistry was performed using a mouse IgG2a-anti human 5-LO/ALOX5 antibody or an IgG2a isotype control (1 µg/ml). One representative example out of 6 independent experiments from 6 different donors is shown. Staining intensity was quantified by a digital computer-assisted method. The percentages of 5-LO/ALOX5 positive cells are shown in scatterplots for monocytes (**B**) and for macrophages (**D**). Data are shown as individual values with medians. Significant differences, as determined by Wilcoxon matched-pairs signed rank test are indicated as follows: *p < 0.05; **B** (n = 6); **D** (n = 6)

Human monocytes from anonymous blood donors were isolated and cultured in growth chambers (chamber tek slides). Macrophages from the same donors were differentiated in the presence of M-CSF and cultured in chamber tek slides in parallel. Cells were either left non-stimulated or stimulated with IFNγ. After removing the medium, the adherent cells on the slide were fixed and subjected to immunocytochemical staining using a mouse IgG2a-anti human 5-LO/ALOX5 antibody. We observed that IFNγ stimulation upregulated the protein expression of 5-LO/ALOX5 in monocytes and macrophages whereas the staining of the non-stimulated or IFNγ-stimulated cells with the respective isotype control showed no signs of unspecific binding (Fig. 4A and C). Summarizing the results of quantification of 5-LO/ALOX5 positive cells using a digital computer-assisted method in 6 independent experiments from 6 different donors, we detected a significant upregulation of 5-LO/ALOX5 protein expression in response to IFNγ in human monocytes and macrophages (Fig. 4B and D).

### Leukotriene-C4-synthase (LTC4S) mRNA expression is upregulated by IL-13 in human monocytes from healthy volunteers (HVs)

**Fig. 5:**
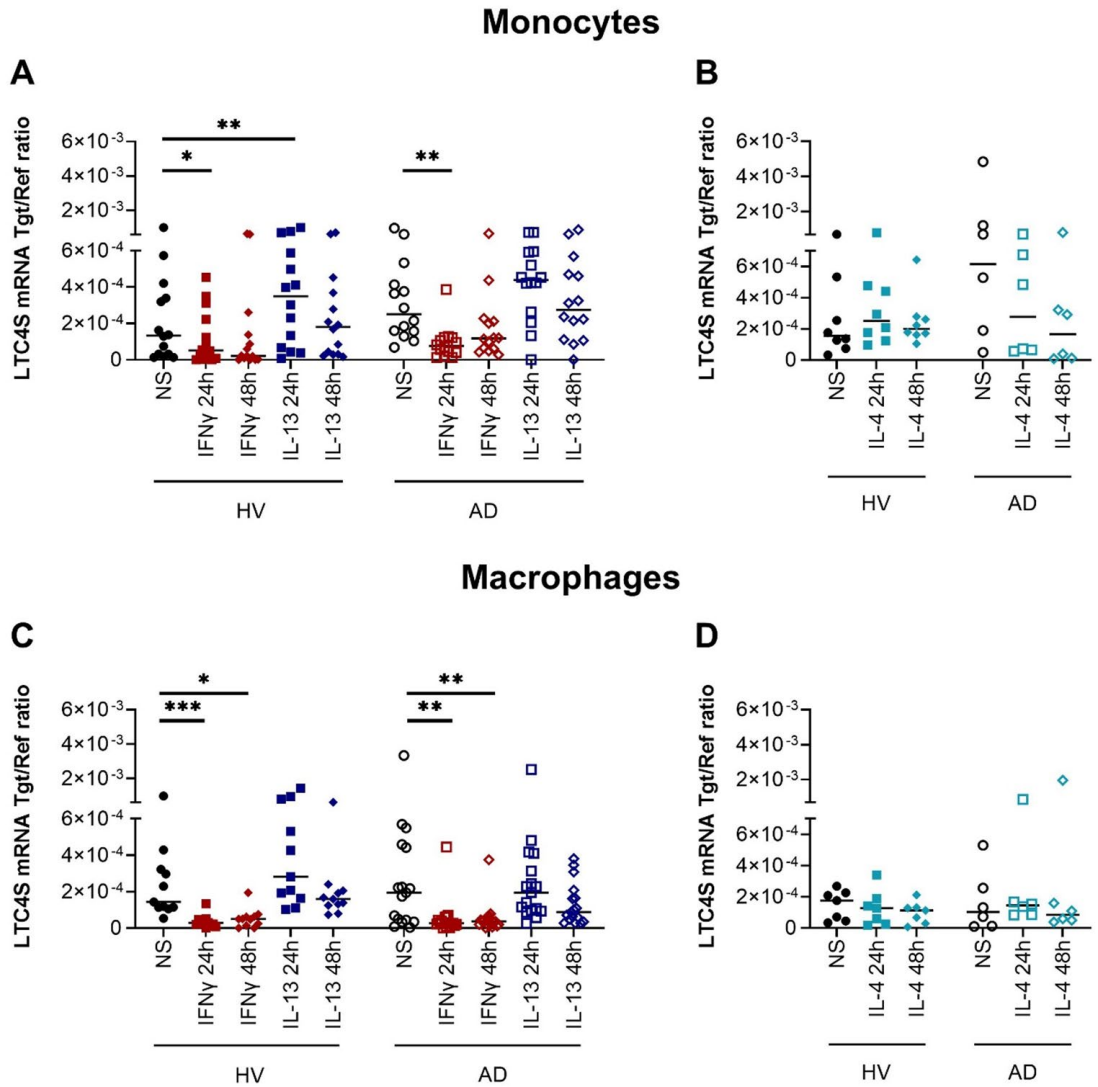
Leukotriene-C4-synthase (LTC4S) mRNA expression is upregulated by IL-13 in human monocytes from healthy volunteers (HVs). Human monocytes (**A**, **B**) and macrophages differentiated in the presence of M-CSF (**C**, **D**) obtained from HVs and from atopic dermatitis (AD) patients were stimulated with IFNγ, IL-13 or IL-4 for 24 and 48 h or left non-stimulated (NS). LTC4S mRNA expression was detected by q-PCR and shown as target/reference (Tgt/Ref) ratio. Data are shown as individual values with medians. Significant differences, as determined by Friedman Dunn´s multiple comparison test are indicated as follows: *p < 0.05; **p < 0.01; ***p < 0.001; **A** (n_HV_ = 14, n_AD_ = 14); **B** (n_HV_ = 8, n_AD_ = 6); **C** (n_HV_ = 11, n_AD_ = 17); **D** (n_HV_ = 7, n_AD_ = 6)

Human monocytes and macrophages differentiated in the presence of M-CSF from HVs and from AD patients were stimulated with IFNγ, IL-13 or IL-4 or left non-stimulated. We analyzed the mRNA expression of LTC4S, the key enzyme for the synthesis of cysteinyl leukotrienes. LTC4S converts LTA4 to LTC4, which is subsequently exported to the extracellular space where it is sequentially converted to LTD4 and LTE4 by peptidases [7]. Unlike the regulation observed for 5-LO/ALOX5, LTC4S mRNA expression was downregulated by IFNγ in monocytes and macrophages from HVs and AD patients. IL-13 significantly upregulated LTC4S mRNA expression in monocytes from HVs and by trend in monocytes from AD patients and in macrophages from HVs (Fig. 5A and C). The stimulation of monocytes and macrophages with IL-4 had no influence on LTC4S mRNA expression (Fig. 5B and D).

## Discussion

CysLTs are potent proinflammatory mediators that play a crucial role in the pathogenesis of various inflammatory diseases, including asthma [23] and AD [7, 8]. Recent studies have shown that elevated levels of CysLTs are present in the skin of AD patients [8]. Moreover, urinary LTE4 levels are significantly increased in moderate and severe AD compared to mild AD and a positive correlation was observed between urinary LTE4 and disease severity scores, absolute eosinophilic counts, and serum total IgE [24, 25].

Functional relevance for the role of CysLTs in the pathogenesis of AD was further supported by studies of murine models of AD, which identified CysLTs as mediators of skin fibrosis and itch [9, 10].

Kiekens et al. described increased numbers of macrophages in lesional skin compared to non lesional skin in AD patients or to healthy skin. Accumulation of macrophages in both acutely and chronically inflamed AD skin highlights their prominent role in the disease pathogenesis [12].

Analysis of single-cell RNA sequencing results in a data set obtained from lesional skin of AD patients of our previous publication [4] revealed higher expression of 5-LO/ALOX5 and LTC4S transcripts in monocytes and macrophages compared to other skin cells, with M1-like macrophages showing elevated 5-LO/ALOX5 transcript levels compared to M2-like macrophages. This observation was further corroborated by our in vitro experiments showing that GM-CSF-derived M1-like macrophages exhibited higher baseline 5-LO/ALOX5 mRNA expression than M-CSF-derived M2-like macrophages and monocytes. These results demonstrate a remarkable concordance between in vivo data from affected patients and in vitro generated data. 5-LO/ALOX5 mRNA expression and the presence of 5-LO/ALOX5 protein in murine bone-marrow-derived macrophages was observed by Sorgi et al. [26]. However, despite its expression the enzyme is mostly inactive showing little to no leukotriene expression. Addition of exogenous AA can activate 5-LO/ALOX5 and induce leukotriene expression [26].

To mimic the inflammatory milieu present in AD skin we examined the expression profiles of 5-LO/ALOX5 and LTC4S in monocytes and monocyte-derived M-CSF macrophages from AD patients and HVs, following stimulation with the AD-associated cytokines IFNγ, IL-4 and IL-13 [1].

We found that 5-LO/ALOX5 and LTC4S mRNA expressions were differentially regulated by the Th1 cytokine IFNγ being overexpressed in chronically inflamed skin in AD and by Th2 cytokines IL-4 and IL-13. 5-LO/ALOX5 mRNA expression was upregulated by IFNγ in monocytes and macrophages from AD patients.

We propose that epigenetic priming may contribute to the upregulation of 5-LO/ALOX5 mRNA expression observed exclusively in cells derived from AD patients. Cells from AD patients often show altered chromatin accessibility and DNA methylation patterns, making certain genes more responsive to pro-inflammatory signals. IFNγ may more effectively induce 5-LO/ALOX5 in AD patient cells because these cells have been primed by chronic inflammation, increasing promoter or enhancer accessibility for IFNγ-driven transcription factors. Also, monocytes and macrophages from AD patients are likely due to systemic inflammatory mediators or repeated exposure to skin-derived signals in an enhanced basal activation state which may sensitize the cells to cytokines like IFNγ [27].

The study from Li et al. [28] provides strong support for differential expression of 5-LO/ALOX5 pathway genes in disease states compared to controls [28]. They observed that elevated 5-LO/ALOX5 expression is linked to oxidative stress and inflammation and can be confirmed in macrophage populations under pathological conditions [28].

IL-4 and IL-13 downregulated 5-LO/ALOX5 mRNA expression in monocytes and macrophages from HVs and AD patients.

The stimulating effect of IFNγ on 5-LO/ALOX5 mRNA expression is in accordance with previous results from Ebert et al. [29] showing an upregulation of 5-LO/ALOX5 mRNA expression in M1 macrophages after a combined stimulation with GM-CSF for 7 d and IFNγ for 48 h, while differentiation of M2 macrophages by M-CSF for 7 d receiving IL-4 in the last 48 h resulted in lower 5-LO/ALOX5 mRNA expression compared to the medium control [29].

We observed that IFNγ upregulated 5-LO/ALOX5 protein both in monocytes and in macrophages from healthy anonymous donors, but no significant increase of mRNA levels in response to IFNγ. This discrepancy may result from translational repression in macrophages under normal/healthy conditions, where mRNA is present, but protein synthesis is blocked until stimulated. This aligns with Schott et al., [30] who showed that proinflammatory mRNAs in macrophages are constitutively expressed but their protein translation is repressed until activation [30]. Thus, IFNγ likely lifts this repression, causing stronger protein upregulation than mRNA increase in macrophages.

The downregulation of 5-LO/ALOX5 mRNA expression in response to IL-4 and IL-13 stimulation in monocyte-derived cells was previously documented by Spanbroek et al. [31]. Their results demonstrated a significant finding: During the process of monocytes-to-DCs transdifferentiation induced by these Th2 cytokines, there was a concurrent downregulation of 5-LO/ALOX5 mRNA expression detectable in developing DCs [31]. However, it is surprising that IL-4 and IL-13, while being key drivers of atopic dermatitis pathology, suppress 5-LO/ALOX5 expression. This may reflect their complex, context-dependent roles on distinct steps of inflammatory pathways. Depending on cell type and environment, IL-4 and IL-13 can either induce or suppress leukotriene-synthesizing enzymes. In macrophages, their suppression of 5-LO/ALOX5 likely highlights their nuanced function in shaping the overall inflammatory milieu.

In human mast cells from AD patients, significantly higher levels of 5-LO/ALOX5 were detected compared to cells from HVs. However, unlike our findings in human monocytes and macrophages, no regulation of mRNA expression for 5-LO/ALOX5 by Th2 cytokines was observed in mast cells of both groups [32]. In contrast to the regulation of 5-LO/ALOX5 expression LTC4S mRNA expression was downregulated by IFNγ in monocytes and macrophages from AD patients and HVs.

Remarkably, we detected a significant upregulation of LTC4S mRNA by IL-13 in monocytes from HVs and by trend only in monocytes from AD patients.

This observation may be explained by the elevated baseline expression of this enzyme in monocytes from patients with AD compared to the baseline expression in cells from HVs. The increased baseline expression of LTC4S in monocytes from AD patients could be driven through endogenous increased exposure of the cells to Th2 cytokines.

The differential regulation of LTC4S, upregulation by IL-13 and no significant regulation by IL-4, although both cytokines signal through the type II IL-4 receptor (IL-4Rα with IL-13Rα1) may be explained that only IL-4 can also use the type I receptor (IL-4Rα with the γc chain). Macrophages express these receptor subunits at varying levels which influences their responsiveness [33]. It is possible that IL-4 elicits a different effect when signaling through the type I receptor compared to the type II receptor, resulting in an overall reduction in LTC4S production.

Furthermore, IL-4 and IL-13 activate distinct patterns of intracellular signaling, and these differences can lead to divergent gene regulation, potentially supporting or opposing LTC4S expression [34].

The differential regulation of the CysLT pathway by Th1 and Th2 cytokines in monocytes and macrophages from AD patients compared to HVs could be attributed to several factors: In acute and chronic phases of AD specific sections of the CysLT pathway in monocytes and macrophages may be selectively activated. Our findings suggest that IFNγ specifically activates the upstream section catalyzed by 5-LO/ALOX5 by upregulating 5-LO/ALOX5 mRNA and protein expression. An example of an upstream product is 5-hydroxyeicosatetraenoic acid (5-HETE), which, after conversion to 5-oxo-ETE [35], attracts eosinophils. This process could potentially explain the increased eosinophil infiltration observed in chronic AD lesions which are associated with higher IFNγ expression in the skin compared to acute lesions, as reported by Kiehl et al. [36].

Our findings indicate that IL-13 primarily enhances the downstream production of CysLTs by upregulating LTC4S mRNA expression. This is of special interest because Hsieh et al. [37] demonstrated that upregulation of LTC4S alone, without concurrent increase in 5-LO/ALOX5 protein levels, is sufficient to boost CysLT production in human mast cells [37].

The elevated levels of LTC4S in monocytes from AD skin can also convert LTA4 derived from human eosinophils or neutrophils during inflammation into CysLTs in a process known as transcellular synthesis [38]. LTC4 has been identified as an itch mediator signaling through the CysLT2 receptor expressed on a subset of sensory neurons in dorsal root ganglia in mouse and humans [10, 11]. The elevated baseline expression in monocytes from AD patients and the upregulation of LTC4S mRNA expression by trend in response to IL-13 suggest that the CysLTs synthesized as consequence of this effect could potentially play a role in the severe pruritus associated with IL-13-driven acute AD lesions [39]. A crucial role for LTC4S has also been proposed in skin fibrosis and inflammation. LTC4 stimulates skin fibroblasts to secrete factors that promote keratinocyte proliferation, as well as collagen deposition leading to epidermal thickening [9].

Our study provides a comprehensive view of the regulation of the cysteinyl leukotriene pathway, covering both key enzymes in monocytes and macrophages, and comparing cells from AD patients with those from HVs for the first time. We demonstrated differential regulation of 5-LO/ALOX5 and LTC4S by Th1 and Th2 cytokines and observed notable overexpression in cells from AD patients.

Although our data suggest that modulation of the CysLT pathway might be therapeutically beneficial, especially given the IFNγ–driven upregulation of 5-LO/ALOX5 in macrophages, the use of leukotriene antagonists is currently inconsistent. Existing studies report variable and often modest improvements with leukotriene antagonists [40, 41]. The translation of our findings into effective therapies in clinical applications could be a task for future research.

## Data Availability

A data availability statement for this journal is provided by the authors, the single-cell RNA sequencing data that were re-analyzed in this study are available in Zhang et al. [4].
